# The BRAF and NRAS status among distinct metastases of malignant melanoma differ significantly independent of tissue origin and temporal occurrence. Possible effect on clinical relevance?

**DOI:** 10.1097/CMR.0000000000000944

**Published:** 2023-12-19

**Authors:** Cameron E. Geiger, Salima Mrabet-Dahbi, Irina Berger

**Affiliations:** a University of Southampton, Klinikum Kassel; b Department of Pathology, Klinikum Kassel, Kassel, Germany

This letter focuses on discrepancies between BRAF and NRAS mutations between different spots of metastases and their effect on clinical outcome.

BRAF has a pivotal role within the mitogen-activated protein kinase (MAPK) signalling pathway, influencing cell cycle progression and proliferation [1]. 70% of melanomas exhibit a mutation in the MAPK pathway, 50% of them include BRAF mutations causing downstream MAPK dysregulation and in consequence permanent cell activation. This led to BRAF emerging as a crucial downstream target for immunotherapy due to its regulative role in the phosphorylation of other kinases within the MAPK pathway [1].

In addition, NRAS mutations are prevalent in approximately 25% of melanoma and display an even more aggressive tumour progression than their BRAF-mutated counterparts, although the role of NRAS mutations only serve as a prognostic factor [2].

The overall survival of patients with BRAF-positive mutant melanoma has improved significantly since the discovery of targeted therapy, although the prognosis of NRAS-mutated patients tends to be far poorer [3]. The only reliable clinicopathological characteristics to determine eligibility for FDA-approved immunotherapy or targeted therapy options (for patients with stage III–IV) are TNM stage and the presence/absence of a BRAF V600E/K mutation [3,4].

Currently, BRAF V600 mutational testing is only available for the primary metastasis of a malignant melanoma, and any other metastasis that arises later will not be tested. Therefore, the possibility of in situ mutations or heterogeneity of metastatic lesions is not considered in the current diagnostic approach. This could lead to a disparity between treatment options for patients whose tested primary metastasis had a BRAF V600 mutation compared to patients who did not.

Forty-six existing tissue samples (34 out of 46 were BRAF/NRAS mutated) of metastatic malignant melanoma from 21 patients that originate from diagnostic testing undertaken at the Klinikum Kassel from January 2015 to September 2022 were analysed. The mutational diagnosis was performed either by PCR and reverse hybridisation using the StripAssay (ViennaLab Diagnostics, Vienna, Austria) or by Idylla (from Biocartis).

Tests for statistical significance were done with the Fishers-Freeman-Halton test due to the small sample size for gender, age group and tissue origin. No statistically significant secondary outcomes were measured.

As shown in Table 1, a total of five patients exhibited a discrepant mutational profile, namely patients no.2, 7, 9, 11 and 21. Consequentially, three cases (patient no.2, 7, 11) exhibited a loss of BRAF status, with the addition of patient no.11 gaining an NRAS mutation status. One case (patient no.9) developed a different BRAF status, and the last case (patient no.21) displayed a gain in NRAS status. The time difference between the primary and secondary/tertiary metastases of the patient cases with a discrepancy in mutational status ranged from simultaneously discovered metastases to metastases that were discovered up to three years apart.

**Table 1:** Mutational status of all metastatic samples within the scope of this study

Patient No.	BRAF mutations			NRAS mutations		
	First	Second	Third	First	Second	Third
1	V600E	V600E	N/A	WT	WT	N/A
2	V600E	WT	N/A	WT	WT	N/A
3	V600K	V600K	N/A	WT	WT	N/A
4	V600K	V600K	N/A	WT	WT	N/A
5	K601E	K601E	N/A	WT	WT	N/A
6	V600K	V600K	N/A	WT	WT	N/A
7	V600E	WT	N/A	WT	WT	N/A
8	V600E	V600E	N/A	WT	WT	N/A
9	V600E	V600K	N/A	WT	WT	N/A
10	V600E	V600E	N/A	WT	WT	N/A
11	V600E	WT	WT	WT	Exon 3: p.Gln61Lys	Exon 3: p.Gln61Lys
12	WT	WT	N/A	Exon 3: p.Gln61Arg	Exon 3: p.Gln61Arg	N/A
13	WT	WT	WT	Exon 3: p.Gln61Lys	Exon 3: p.Gln61Lys	Exon 3: p.Gln61Lys
14	WT	WT	N/A	Exon 3: p.Gln61Arg	Exon 3: p.Gln61Arg	N/A
15	WT	WT	N/A	Exon 3: p.Gln61Lys	Exon 3: p.Gln61Lys	N/A
16	WT	WT	N/A	Exon 3: p.Gln61Arg	Exon 3: p.Gln61Arg	N/A
17	WT	WT	N/A	WT	WT	N/A
18	WT	WT	N/A	WT	WT	N/A
19	WT	WT	N/A	WT	WT	N/A
20	WT	WT	WT	WT	WT	WT
21	WT	WT	WT	WT	Exon 2: p.Gly12Asp	Exon 2: p.Gly12Asp

WT, wild-type.

The results showed a significant discrepancy regarding the BRAF and NRAS status of different metastatic sites in 5 of 21 (23.8%) patients. Our findings are also supported by a previous study on BRAF and NRAS mutation frequencies among primary tumours and their paired metastasis describing a 15% discrepancy in mutational status [5]. This indicates an unstable mutation status of metastases. On the one hand, this can be explained by the primary tumour’s hereditary nature, in which various tumour cell subsets a priori have various survival advantages regarding metastasis. On the other hand, regarding metastases that arise with a temporal lag, the possibility that the mutations appear in the tumour cells during the process of metastasis or are lost from the still unknown mechanisms regulating tumour progression cannot be excluded. We assume a heterogenous nature of the primary tumour, in which different cell groups develop different mechanisms of tumour progression or metastatic riveting, which appear to exist independently of each other.

We suspect that the proportion of patients who are non-responders to BRAF inhibitor treatment contain patients with melanoma metastases that do not share the same BRAF status, wherein beside the BRAF mutated metastases further tumour spots with BRAF wild-type are present. As targeted molecular therapy is not effective for metastases without a BRAF mutation, this treatment would only have a partial response. A poor response to targeted molecular therapy in the case of a discrepancy between different spots of metastases can be rationalized.

Overall, a recommendation on the frequency of molecular testing of BRAF and NRAS status can be approximated to include at least two metastases, if not all metastases that arise over the course of tumour progression. To provide optimal patient care, these discrepancies need to be taken into consideration clinically, to encourage the mutational diagnosis of each newly arising metastasis.

## Acknowledgements

The authors would like to thank the staff at the laboratory for molecular pathology of the Klinikum Kassel, Germany, for dedicating their time to supervise the mutational diagnosis. They would also like to thank the statistical support provided by the University of Southampton, UK, and the Kassel School of Medicine, Germany.

### Conflicts of interest

There are no conflicts of interest.
